# Associations Between Screen Time and Mindfulness and Eating Behaviors Among Turkish School-Aged Children: A Cross-Sectional Study

**DOI:** 10.3390/children12060696

**Published:** 2025-05-29

**Authors:** İlayda Temizarabacı, Gizem Köse, Murat Baş, Ina Nehring

**Affiliations:** 1 Health Science—Prevention and Health Promotion, School of Medicine and Health, Technical University of Munich, 80333 Munich, Germany; 2 Department of Nutrition and Dietetics, Health Sciences Faculty, Acıbadem Mehmet Ali Aydınlar University, 34752 İstanbul, Türkiye; gizem.kose@acibadem.edu.tr (G.K.); murat.bas@acibadem.edu.tr (M.B.); 3 Social Peadiatrics, School of Medicine and Health, Technical University of Munich, 81377 Munich, Germany; ina.nehring@tum.de; 4 German Center for Child and Adolescent Health (DZKJ), Partner Site Munich, 80636 Munich, Germany

**Keywords:** screen time, mindfulness, mindful eating, diet quality, school-aged children

## Abstract

**Background/Objectives:** Increasing screen time in childhood has been suggested to impact physical health, eating behaviors, and well-being. This study investigated how screen time affects mindfulness, mindful eating, and diet quality in Turkish adolescents aged 9–12 years. **Methods:** One hundred thirty-seven participants completed surveys on anthropometry, screen time, the Godin Leisure-Time Exercise Questionnaire, the Mediterranean Diet Quality Index, the Mindful Eating Questionnaire for Children, and the Child and Adolescent Mindfulness Measure. **Results:** The average screen time was 4.43 ± 2.37 h/day. Significant negative correlations emerged between screen time and mindfulness (r = −0.471, *p* < 0.001), as well as between screen time and diet quality (r = −0.244, *p* < 0.05). A regression analysis revealed significant associations only for mindfulness (B = −0.158, *p* < 0.001) and age (B = 0.636, *p* = 0.002). **Conclusions:** Higher mindfulness and younger age correlated with lower screen time, suggesting mindfulness interventions in schools may reduce screen use.

## 1. Introduction

Today’s children and adolescents live in a media-saturated world and have easier access to mass media than previous generations. The American Academy of Pediatrics (AAP) recommends restricting children’s television viewing and other recreational screen time to less than 2 h per day [1]; however, the World Health Organization (WHO) states that there is currently insufficient evidence to establish a specific threshold for sedentary or recreational screen time [2]. Despite these recommendations and uncertainties, the estimated total media use of Western adolescents is 40 h per week or 5 h per day [3]. One study involving high school students in Türkiye estimated their average screen time at 3.41 h/day, which was above the recommended duration but lower than the average for developed countries [4]. The COVID-19 pandemic greatly affected the screen habits of children and adolescents, as almost 25 million Turkish students received distance education [5]. In one study from Türkiye that examined the impact of COVID-19 on children’s screen time, 71.7% of parents reported that their children’s screen time increased during the pandemic, reaching approximately 6.42 h/day [6]. Although the pandemic has ended, habits acquired during this period are likely to have permanently affected the screen consumption of children and adolescents.

Post-1980 epidemiological research indicates that time spent in front of the screen is associated with the risk of future obesity [7]. According to one study conducted on children aged 9–10 years old, screen time exceeding 3 h/day was associated with greater obesity than less than 1 h of screen time [8]. In 2200 randomly selected 9- to 16-year-old Australians, overweight and obesity were found to be even more strongly associated with screen time than physical activity [9]. Another large-scale study involving 3275 children aged 5 to 14 found that longer durations of TV viewing were associated with increased odds of being overweight or obese and that children who watched two or more hours of TV daily were more than twice as likely to exhibit dietary patterns linked to increased obesity, primarily due to exposure to food advertisements [10]. While that study emphasized the role of advertising, other contributing factors—such as decreased physical activity, increased food consumption in front of screens, and disrupted sleep patterns—are generally explained as further causes for the correlation [7,11,12]. Studies are increasingly examining new approaches such as “mindful eating” and “mindfulness”, especially their effect on dietary habits and the prevention of obesity. Recognition of the significance of mindful eating is increasing, particularly in response to the largely unsuccessful treatments offered for the maintenance of weight loss [13].

Self-awareness and the regulation of attention and emotions have all been proposed as key components associated with mindfulness, which is an individual’s capacity to maintain present-moment awareness with acceptance. These mechanisms may help individuals resist automatic behaviors, such as excessive screen engagement, which are often driven by habitual or emotionally reactive responses. [14]. Conversely, increased screen time has been linked to attentional lapses and cognitive overload [15], which can, in turn, undermine mindfulness. Therefore, the relationship between mindfulness and screen use may be reciprocal, potentially mediated by executive functioning and impulse control capacities.

Mindfulness can also have an impact on changes in transformative health behavior [16]. Mindfulness practices are expected to lead to positive changes in mothers and their children’s nutritional behaviors [17]. A meta-analysis reported a small but consistent association between trait mindfulness and health-promoting behaviors, and this link was supported by another study, which found that individuals with higher mindfulness were less likely to choose unhealthy foods [18,19]. Studies on the potential for mindfulness to prevent or reduce overeating that leads to a higher body mass index (BMI) among adolescents and young adults have shown promising results [20,21], indicating the need for scientific research to understand the underlying mechanisms.

Mindfulness practices in the field of eating behavior give rise to another new concept: mindful eating. Mindful eating focuses on how and why eating behavior occurs rather than what is eaten and involves internalizing physical hunger and satiety signals [22]. The use of mindful eating interventions has expanded in recent years to include children. The aim is to draw attention to the sensory aspects of eating [23]. A study involving 10- to 12-year-old adolescents identified two mindfulness exercises (mindful breathing and mindful raisin eating) as being particularly useful in encouraging adolescents to try new foods [24]. However, a 10-session intervention study conducted on preschool children suggested the effect was minor: although the intervention group was more willing to try new foods during mindful eating exercises, the difference did not rise to the level of significance [25].

Despite the association between increased screen time and the decreased quality of children’s diets [26,27], few studies have examined mindfulness and mindful eating, which are likely to interact with screen time and diet quality [28]. This study was designed to assess the hypothesis that diet quality, mindfulness, and mindful eating levels decrease in school-aged children with increasing screen time levels. As a secondary outcome, the study will also include the participants’ BMI for age and exercise levels to further investigate correlations with screen time. This research is intended to identify the possible relationships between screen time and diet quality, mindfulness, and mindful eating levels among school-aged children and provide valuable information to protect them from the possible harmful effects of excessive screen time, with the aim of promoting a healthy lifestyle and thus preventing possible health problems. This integrative approach—combining behavioral, psychological, and dietary variables—provides a novel perspective on screen-related health risks in school-aged children.

## 2. Materials and Methods

### 2.1. Participants and Recruitment

Students from three private schools located in the city of Bursa in northwestern Türkiye were selected to participate in the study; these institutions were chosen based on accessibility, willingness to participate, and their relatively homogeneous socioeconomic student populations, which may enhance the generalizability of the findings. Written permissions were obtained from the principals of the three schools. The written informed consent form was given to all students attending the fourth, fifth, and sixth grades, as well as to their parents. After receiving their families’ permission, student volunteers filled out questionnaires in the presence of a school psychologist during the psychological counseling hours at their schools. Five weeks were allocated for data collection, starting in early September 2023.

Participants were suitable for the study if they met the following inclusion criteria: they were registered students in the fourth, fifth, and sixth grades of the above-named private schools and had no chronic disease. Inclusion in the study required both parental approval and voluntary participation from the students themselves. All students in the three participating schools within the age group corresponding to the study population were invited to participate without any form of discrimination.

The exclusion criteria are as follows: chronic illnesses that might potentially affect the diet or preexisting medical conditions, as these factors could introduce confounding variables that might affect the research outcomes [29]. Volunteers who adhered to a regular nutrition program or dietary plan without illnesses were also excluded, as this could influence their dietary patterns. Furthermore, individuals taking medications that had a significant impact on their diet or nutritional intake were not included in the study. By applying these eligibility criteria, the researchers sought to ensure the suitability of participants and enhance the validity and reliability of the collected data. Among the participants, two celiac patients and one epilepsy patient were excluded from the study because of their particular nutritional requirements. A total of 137 participants (67 girls and 70 boys; mean age = 10.18 ± 0.92) were eligible for the study.

### 2.2. Measures

#### 2.2.1. Demographics and Screen Time

Part A of the questionnaire includes the student’s age, sex, height, and weight information. Additionally, it asks about the presence of any chronic health condition, regular medications taken, and whether the student follows a special dietary program. Part B of the questionnaire contains questions aimed at measuring screen habits adopted from SCREENS-Q [30]. The survey asks for simple yes/no responses regarding the student’s ownership of a personal phone, parental screen limitations, the need for prior permission from parents for screen usage, and the practice of using screens while eating.

Students were asked to specify the number of electronic devices present in their household, including laptops, desktop computers, tablets/iPads, smartphones, televisions, and gaming consoles. Lastly, they indicated the amount of time they spent in five predefined categories (0 to 4+ hours) on both weekdays and weekends. The categories were as follows: (1) watching movies, series, or videos; (2) playing video games; (3) video calls; and (4) social media platforms.

To calculate the average screen time, the total weekend and total weekday screen times were calculated and divided into two. The categories of average screen time were as follows: low for 0–2 h/day, medium for 2–4 h/day, and high for >4 h/day.

Both the students and their parents self-reported height (m) and body weight (kg) measurements. In nine surveys in which the parent and child reported different data, the parent’s statement was accepted, given that the participants were young and did not know their exact weight and height. Participants’ BMI was calculated using the WHO AnthroPlus software (version 1.0.4), which is intended for the global application of the WHO Reference 2007 for 5- to 19-year-olds to monitor the growth of school-aged children and adolescents [31]. The sex- and age-specific standard deviation scores (SDS) (i.e., BMI for age) obtained from the AnthroPlus were also used to define overweight and thinness (including thinness: <−2SD; overweight: >+1SD and obesity: >+2SD) [32].

#### 2.2.2. Leisure Time Exercise

The participants’ exercise time was measured using the self-reported Godin Leisure-Time Exercise Questionnaire (GLTEQ) [33]. The scale, which was created by Godin and Shephard in 1985, is intended to measure the exercise activity of individuals during their leisure time. The Turkish version of GLTEQ was found to be a valid and reliable questionnaire for determining the leisure time exercise levels of 10- to 17-year-old students [34]. The scale asks students whether they have spent at least 15 min of free-time on physical activity over the last seven days and attempts to determine strenuous/moderate/mild-light exercises with a total weekly leisure time activity score. The Godin scale score interprets 24 units or more as “active”, 14–23 units as “moderately active”, and fewer than 14 units as “insufficiently active/sedentary”.

#### 2.2.3. Diet Quality

The Mediterranean Diet Quality Index (KIDMED) [35], consisting of 16 questions with yes/no answers, was used to determine the general diet quality of students. The index includes self-reported questions, such as whether one consumes vegetables and fruit on a daily basis, goes to fast food restaurants, or skips breakfast. Questions with a negative connotation (items 6, 12, 14, and 16), such as those about skipping breakfast or eating sweets and candies several times per day were scored as −1, while questions with a positive connotation, such as those about using olive oil at home or consuming fish regularly, were scored as +1 for a “yes” answer. All questions with a “no” answer was scored as 0. The scale, which was adapted into Turkish in 2019, was suitable for use in Türkiye [36]. The total KIDMED scores range from 0 to 12 and are classified as follows: ≥8 points, good (optimal MedDiet); 4–7 points, average; and ≤3 points, poor.

#### 2.2.4. Mindful Eating

The Mindful Eating Questionnaire for Children (MEQ-C) [37], which consists of 12 questions and is suitable for use between the ages of 8 and 11, was used to determine the mindful eating scores of students. MEQ-C, a self-report questionnaire consisting of two subscales (mindless eating and awareness), was adapted into Turkish and found appropriate for use with Turkish children [28]. The “Mindless eating” subscale has eight related questions, and higher scores on the 4-point Likert scale indicate less mindful eating, whereas “Awareness” has four related questions, and higher scores indicate increased awareness. The scores of the items in each subdimension are summed up and then divided by the number of items to obtain a total score for the two subdimensions separately. The Cronbach’s alpha coefficient was 0.82 for the mindless eating subscale and 0.80 for the awareness subscale [28].

#### 2.2.5. Mindfulness

The Child and Adolescent Mindfulness Measure (CAMM) [38], consisting of eight questions suitable for 10- to 17-year-olds, was used to determine students’ self-reported level of mindfulness. All items were reverse-coded using a 5-point Likert scale ranging from 0 (not true) to 4 (always true). The total score on the CAMM was calculated by summing up the responses to all eight items, with higher scores indicating a higher level of mindfulness in the child or adolescent. The scale was adapted to Turkish and found to be valid for Turkish children and adolescents, with a Cronbach’s alpha of 0.79 [39].

### 2.3. Statistical Analysis

An a priori power analysis was conducted using G*Power version 3.1.9.2 to determine the minimum sample size required to test the study hypothesis. The required sample size to achieve 90% power for detecting a medium effect, with a significance criterion of α = 0.05, was N = 97 for the chi-square goodness-of-fit test.

Descriptive statistics for categorical variables, including demographic characteristics, were presented as frequencies and percentages, while those for participants were presented as means ± standard deviations ($\overset{-}{X}$ ± SD) and ranges (min–max). The relationships between the variables were assessed using the Pearson correlation coefficient. If not otherwise specified, the statistical significance level was set at *p* < 0.05 for all calculations and interpretations. Regression analysis was used to evaluate the relationship between screen time and diet quality, mindfulness, mindful eating, sex, age, and exercise levels. A stepwise regression analysis was conducted to examine the relationship between mindless eating and BMI for age. The assumptions of the normality of the residuals (Q-Q plots of residuals and Kolmogorov–Smirnov and Shapiro–Wilk Normality tests), the presence of linear relationships between time and the independent variables (correlation matrix), the lack of multi-collinearity (correlations and variance inflation factor), and the homoscedasticity (scatter plot of residuals) were verified. Statistical analyses were performed using the Statistical Package for the Social Sciences (version 23; IBM Corp., Armonk, NY, USA).

## 3. Results

According to the WHO’s BMI-for age classification, 62.8% of the participants (n = 137) were of normal weight, while nearly one in four (24.8%) were classified as overweight. Among the participants, 64.2% reported owning a personal phone; a substantial proportion also reported parental controls, such as screen limits (60.6%) and needing permission to use screens (54.7%). Most students (63.5%) did not use screens during meals. According to the KIDMED scores, 62.0% of participants were classified as having average dietary quality, and only one-quarter (25.6%) met the criteria for a good Mediterranean diet. According to leisure time exercise scores, more than 90% were at least moderately active. Importantly, nearly half of the participants (46.0%) were in the high-screen group, with a screen time of more than 4 h/day, and only 20.4% of them were in the low-screen group, with a screen time of 0–2 h/day. The demographic characteristics of the participants are shown in Table 1.

The screen time and anthropometric demographics of the participants are shown in Table 2. The mean age of the participants was 10.18 ± 0.92, and the mean BMI was 17.64 ± 2.64 kg/m^2^. The average daily screen time was 4.43 ± 2.37 h, with a considerable range from 0.50 to 9.50 h per day. Screen use was higher on weekends (6.19 ± 3.08 h/day) than on the weekdays (2.66 ± 2.12 h/day), and watching films or videos was the most commonly reported activity across both periods. Notably, twenty-five participants reported no screen use on weekdays. The average score for mindfulness (CAMM) was 22.59 ± 5.95. The average score of the awareness subscale of MEQ-C was 2.97 ± 0.65, while for the mindless eating subscale, it was 1.75 ± 0.53.

Table 3 presents the correlation coefficients of the variables. Mindfulness was significantly positively correlated with diet quality (r = 0.331, *p* < 0.001) and significantly negatively correlated with mindless eating (r = −0.370, *p* < 0.001), average screen time (r = −0.471, *p* < 0.001), and BMI (r = −0.196, *p* = 0.022). The average screen time was also significantly negatively correlated with diet quality (r = −0.244, *p* = 0.004) and BMI (r = −0.196, *p* = 0.022). Notably, leisure time exercise was not significantly correlated with any of the measured variables (*p* > 0.05).

Table 4 presents the results of the linear regression analysis for the outcome variable of average screen time. Among all predictors, only mindfulness (B = −0.158, *p* < 0.001) and age (B = 0.636, *p* = 0.002) were significantly associated with average screen time. Specifically, lower mindfulness scores and older age predicted higher screen time. Other included variables were not significantly associated with average screen time (*p* > 0.05). Table 5 displays the fully adjusted regression model predicting BMI for age. In the regression model without adjustment for confounders, there was a significantly positive association between mindless eating and BMI for age (*p* = 0.025). The fully adjusted model did not show any significant associations between BMI for age and the included variables (*p* > 0.05) (Table 5).

## 4. Discussion

In this study investigating the relationship between screen time and mindfulness, diet quality, and mindful eating levels, high screen time was associated with low mindfulness levels but not with mindful eating and diet quality among 9- to 12-year-olds in Turkish private schools. A significant association was identified between mindless eating and BMI for age. However, this association faded after adjustment for potential confounders. None of the examined variables were significantly correlated with leisure time exercise.

The fully adjusted linear regression model yielded significant coefficients for mindfulness and age: −0.158 and 0.636, respectively. This suggests that for each one-unit increase on the overall mindfulness scale, the average screen time decreases by 0.158 units, or 9.48 min/day, holding all other variables constant, or vice versa. Therefore, the regression analysis findings indicate that higher levels of mindfulness and younger age were associated with lower levels of screen time among our participants.

The average screen time in the study population was 4.43 ± 2.37 h per day; both weekday (2.66 ± 2.12 h/day) and weekend (6.19 ± 3.08 h/day) average screen time for participants were higher than the AAP’s recommended levels of about 2 h per day. The activity on which participants spent the most screen time, both on weekdays and weekends, was watching movies/videos, followed by playing games. A study conducted on 10- to 12-year-olds in public schools in Türkiye in 2012 determined that 47.9% of adolescents spent two hours/day or more in front of a screen [40]. In our study, this rate was much higher, at 79.6%, for the same age group in private schools. Our finding is consistent with that of a study that found that children from private schools in Türkiye spend more time in front of screens than those from public schools [4]. According to the 2022/23 academic year-end formal education statistics published by the Ministry of National Education, approximately 8% of students were educated in private schools [41]. This population is expected to consist of members of high-socioeconomic status (SES) families. Therefore, not only will they have more personal technological devices, they will also likely have greater access to technologies such as the internet than their public counterparts [4]. A systematic review and meta-analysis has identified a strong and consistent inverse relationship between SES and total screen time in high-income countries; however in to low-to-middle-income countries, a positive association has found between SES and screen time [42], which is confirmed by our results.

The present study did not find a significant relationship between screen time and BMI or BMI for age, in contrast to the study in Türkiye that identified a relationship between increased screen time and obesity among adolescents [35] and despite a meta-analysis of 16 studies that found that the risk of overweight/obesity in adolescents increases with a screen time of ≥2 h/day, compared to <2 h/day [43]. This might be because 73.7% of the participants are in the active category, as weekly sports instruction is compulsory for students attending private schools. However, another Turkish study also found no difference between the overweight–obese group and the normal–thin adolescent group in terms of TV viewing and computer use time, which is consistent with the results of the present study [44]. Additionally, a cross-sectional study conducted on children in six European countries found no significant relationship between children’s BMI and their screen time [45]. Furthermore, a negative correlation between diet quality and screen time was identified but could not be confirmed in the adjusted regression, suggesting that screen time might not be the main risk factor for lower diet quality, at least among this population.

With regard to the negative association between mindfulness and screen time, we found only a few studies that investigated this relationship. Although one study showed no relationship between mindfulness and screen time in children aged 8–13 [46], another study highlighted the negative correlation between time spent online and mindfulness among adolescents [47]. Indeed, studies on mindfulness and screen time involving children or adolescents are very sparse. However, some studies have examined screen time and “attention”, which is considered a basic component of mindfulness; one systematic review identified a negative relationship between screen time and attention among children and adolescents of a comparable age group [48].

A lack of mindfulness is arguably both a consequence and a predictor of high screen time [49]. One study revealed that self-esteem and social anxiety mediate the effects of mindfulness on reducing the compulsive use of social networking sites [50]. Among adolescents, increased mindfulness significantly lowers the likelihood of preferring online social interactions and using the internet as a tool for mood regulation [51]. Consequently, one can plausibly assert that the correlation between mindfulness and screen time is not solely directed. The preceding discussion raises two possibilities. First, increased screen time might lead to decreased attention and mindfulness levels. For example, prior research suggests that smartphone usage is linked to higher cognitive failure; thus, it is possible that heavy smartphone use increases cognitive failure, which in turn reduces mindfulness [52]. Second, people with a high level of mindfulness tend to avoid obstacles, such as screens, that prevent them from focusing on the moment with the mediating role of self-control [53].

We also found that diet quality has a significantly negative correlation with screen time but a positive correlation with mindfulness. This result is consistent with another study that investigated the screen time and dietary habits of 177,091 children aged 8–17 using KIDMED, which found longer screen time to be associated with higher odds of unhealthy dietary habits [26], confirming a multifactorial unhealthy lifestyle. However, unlike other studies, our study found no correlation between diet quality and BMI [54]. Previous studies have concluded that for our population’s age group, mindfulness is unrelated to both vegetable and fruit intake [46]. However, our study’s finding of significantly positive relationships between mindfulness and diet quality may be attributed to the more complex Mediterranean diet that subjects adhered to, rather than the consumption of fruits and vegetables.

Previous studies have found that mindless eating scores are higher and awareness scores are lower among obese/overweight children and concluded that mindless eating habits were more common, especially among obese children [55]. Our study identified a positive correlation between BMI for age and mindless eating but no correlation with awareness. However, in the adjusted regression model, none of the included variables had statistically significant effects on BMI for age. This suggests that other factors not included in our model, such as genetics, sleep patterns, or daily caloric intake, may have a stronger influence on BMI for age among our participants. Additionally, SES may be another factor affecting BMI in our study; 11 studies have consistently found that children from higher socioeconomic backgrounds in developing countries tend to have higher rates of overweight/obesity [56].

Our study did not find significant associations between school-aged children’s screen time and exercise levels. Studies have been inconclusive about the relationship between screen time and exercise levels. One study conducted in Brazil showed that the prevalence of high screen time was significantly higher among insufficiently active adolescents [57]; however, another study found no significant association between the amount of time children spend in front of the television and their exercise levels [58]. We assume that the generally high level of exercise among these private school students might reduce the ability to detect a significant correlation.

To the best of our knowledge, this is the first study to examine the associations between screen time and mindfulness, mindful eating, and diet quality in school-aged children attending private schools. All the scales used were adapted to Türkiye, and their validity was established. In addition, the number of people required for the research specified in the power analysis was provided. This study only included students from private schools with higher SES levels and greater access to technological devices, making it difficult to generalize the results. Additionally, due to the age group, many surveys were not returned, possibly because students forgot to deliver the consent forms, which may have led to a mild selection bias favoring families that are more engaged in monitoring children’s habits. Furthermore, given the young age of the participants, there is a possibility that some children may have had difficulty comprehending certain questionnaire items, and relying solely on self-reported data may have introduced bias, especially for physical activity and body composition. Although variables such as parental screen time limits, permission to use screens, and screen use during meals were collected to capture aspects of parental control, they were not included in the main regression analyses, which represents an additional limitation. Moreover, other unmeasured family-based factors—such as parenting style, parental stress, and household screen norms—may also influence both mindfulness and screen use and should be explored in future studies. Lastly, the cross-sectional design constrains the ability to draw causal inferences about the relationships examined. Although correlations were observed between mindfulness levels and screen time, the possibility of bidirectional effects makes it difficult to determine which factor exerts a greater influence on the other.

## 5. Conclusions

The relationship between mindfulness practices and screen time among school-aged children is a new and promising area for research. Although our study did not align with research that shows a relationship between screen time and BMI/obesity, we still encourage children and adolescents to avoid exceeding the recommended limits for screen time. Our findings indicate that higher screen time is associated with lower mindfulness levels among school-aged children. Although it remains unclear if the correlation between mindfulness and screen time is bidirectional, integrating mindfulness practices into schools could help students to reduce their screen time, encourage healthier habits, and promote overall well-being.

## Figures and Tables

**Table 1:** Demographic variables of participants (n = 137).

	n	%
**Sex**		
Male	70	51.1
Female	67	48.9
**BMI for age**		
Underweight	6	4.4
Normal weight	86	62.8
Overweight	34	24.8
Obesity	11	8.0
**Mobile phone owning**		
Having a mobile phone	88	64.2
Not having a mobile phone	49	35.8
**Screen limitation**		
Having a screen limitation	83	60.6
Not having a screen limitation	54	39.4
**Permission to use a screen**		
Must have permission	75	54.7
No need for permission	62	45.3
**Screen use during mealtime**		
Use the screen during meals	50	36.5
Do not use any screen during meals	87	63.5
**Screen time**		
Screen time (0–2 h/day)	28	20.4
Screen time (2–4 h/day)	46	33.6
Screen time (>4 h/day)	63	46.0
**KIDMED Mediterranean diet quality**		
Poor	17	12.4
Average	85	62.0
Good	35	25.6
**Leisure time exercise**		
Not active	13	9.5
Moderately active	23	16.8
Active	101	73.7

**Table 2:** Descriptive statistics of participants (n = 137).

	Mean ± SD	Min	Max
**Age (year)**	10.18 ± 0.92	9.0	12.0
**Height (cm)**	146.11 ± 7.37	125.0	166.0
**Body weight (kg)**	37.94 ± 7.50	24.0	60.0
**Height for age z-score**	1.07 ± 0.99	−2.13	4.27
**BMI for age z-score**	0.29 ± 1.20	−2.65	3.26
**BMI (kg/m^2^)**	17.64 ± 2.64	12.4	25.3
**People living in the household**	3.77 ± 0.74	2.00	7.00
**Weekend screen time** **(hours/day)**			
Weekend/watching film/video	2.25 ± 1.12	0	4.00
Weekend/playing games	1.90 ± 1.24	0	4.00
Weekend/video call	0.72 ± 0.71	0	2.00
Weekend/social media	1.32 ± 1.51	0	5.00
Weekend screen time total	6.19 ± 3.08	0	13.0
**Weekday screen time** **(hours/day)**			
Weekday/watching film/video	0.99 ± 0.75	0	2.00
Weekday/playing games	0.70 ± 0.83	0	4.00
Weekday/video call	0.35 ± 0.48	0	1.00
Weekday/social media	0.62 ± 0.85	0	4.00
Weekday screen time total	2.66 ± 2.12	0	7.00
**Average screen time** **(hours/day)** ((Weekday + Weekend-day)/2)	4.43 ± 2.37	0.50	9.50
**Mindful eating (MEQ-C)**			
Awareness (ME Subscale)	2.97 ± 0.65	1.25	4.00
Mindless eating (ME Subscale)	1.75 ± 0.53	0.88	3.25
**Mindfulness (CAMM**)	22.59 ± 5.95	6.0	32.0

BMI = Body mass index. MEQ-C = Mindful Eating Questionnaire for Children. CAMM = Child and Adolescent Mindfulness Measure.

**Table 3:** Correlation of scales and BMI variables.

		Mindfulness (CAMM)	Awareness (MEQ-C Subscale)	Mindless Eating (MEQ-C Subscale)	Diet Quality	Leisure Time Exercise	Average Screen Time ^a^ (Hours)	Weekday Screen Time (Hours)	Weekend Screen Time (Hours)	BMI	BMI for Age
Mindfulness (CAMM)	r		0.05	−0.370 **	0.331 **	0.014	−0.471 **	−0.410 **	−0.443 **	−0.196 *	−0.135
	*p*		0.563	<0.001	<0.001	0.872	<0.001	<0.001	<0.001	0.022	0.115
Awareness (MEQ-C**Subscale)	r			0.01	0.13	0.092	−0.042	−0.035	−0.041	−0.047	−0.025
	*p*			0.905	0.13	0.287	0.623	0.684	0.634	0.583	0.768
Mindless eating (MEQ-C Subscale)	r				−0.143	0.163	0.16	0.132	0.156	0.132	0.191 *
	*p*				0.095	0.056	0.061	0.125	0.069	0.123	0.025
Diet Quality	r					0.112	−0.244 *	−0.222 *	−0.221 *	−0.062	−0.026
	*p*					0.191	0.004	0.009	0.009	0.469	0.761
Leisure time exercise	r						−0.015	0.041	−0.051	0	0.047
	*p*						0.861	0.633	0.551	0.997	0.589
Average Screen Time ^a^ (hours)	r							0.868 **	0.940 **	0.122	0.085
	*p*							<0.001	<0.001	0.156	0.321
Weekday Screen Time	r								0.647 **	0.046	0.021
	*p*								<0.001	0.594	0.812
Weekend Screen Time	r									0.156	0.117
	*p*									0.069	0.172
BMI	r	−0.196 *	−0.047	0.132	−0.062	<0.001	0.122	0.046	0.156		0.950 **
	*p*	0.022	0.583	0.123	0.469	0.997	0.156	0.594	0.069		<0.001
BMI for age	r	−0.135	−0.025	0.191 *	−0.026	0.047	0.085	0.021	0.117	0.950 **	
	*p*	0.115	0.768	0.025	0.761	0.589	0.321	0.812	0.172	<0.001	

CAMM: Child and Adolescent Mindfulness Measure. MEQ-C: Mindful Eating Questionnaire for Children. * *p* < 0.05, ** *p* < 0.001. ^a^ (Weekday + weekend screen time)/2 (hours).

**Table 4:** Adjusted linear regression model for the outcome “average screen time”.

	Unstandardized Coefficients		Standardized Coefficients		
	B	Std. Error	β	t	*p*
Constant *	1.941	2.627	-	0.739	0.461
Awareness (ME Subscale)	−0.211	0.276	−0.058	−0.764	0.446
Mindless eating (ME Subscale)	−0.033	0.376	−0.007	−0.088	0.930
Mindfulness	−0.158	0.34	−0.397	−4.640	<0.001
Diet Quality (KIDMED)-Total	−0.082	0.091	−0.072	−0.900	0.370
Leisure time exercise	−0.003	0.016	−0.014	−0.177	0.860
Age	0.636	0.200	0.246	3.176	0.002
Sex	0.547	0.369	0.116	1.482	0.141

Adjusted R^2^ = 0.259. * Dependent variable: average screen time (weekday + weekend screen time)/2.

**Table 5:** Regression analysis for the outcome “BMI-for age”.

	Unstandardized Coefficients		Standardized Coefficients		
	B	Std. Error	β	t	*p*
Constant *	−0.160	0.991		−0.162	0.872
Awareness (ME Subscale)	−0.040	0.161	−0.022	−0.248	0.805
Mindless eating (ME Subscale)	0.356	0.212	0.157	1.680	0.095
Mindfulness total	−0.010	0.20	−0.050	−0.495	0.622
Screen group	0.075	0.143	0.049	0.529	0.598
Diet Quality (KIDMED) group	−0.055	0.180	−0.028	−0.308	0.758
Exercise group	0.045	0.162	0.024	0.277	0.782

* Dependent Variable: BMI for age. ME: mindful eating.

## Data Availability

The anonymized data presented in this study are available upon request from the corresponding author due to confidentiality agreements with participants.

## Abbreviations

The following abbreviations are used in this manuscript:

AAP	American Academy of Pediatrics
WHO	World Health Organization
BMI	Body Mass Index
SD	standard deviation
GLTEQ	Godin Leisure-Time Exercise Questionnaire
KIDMED	The Mediterranean Diet Quality Index
MEQ-C	The Mindful Eating Questionnaire for Children
CAMM	The Child and Adolescent Mindfulness Measure
ME	mindful eating
SES	socioeconomic status
